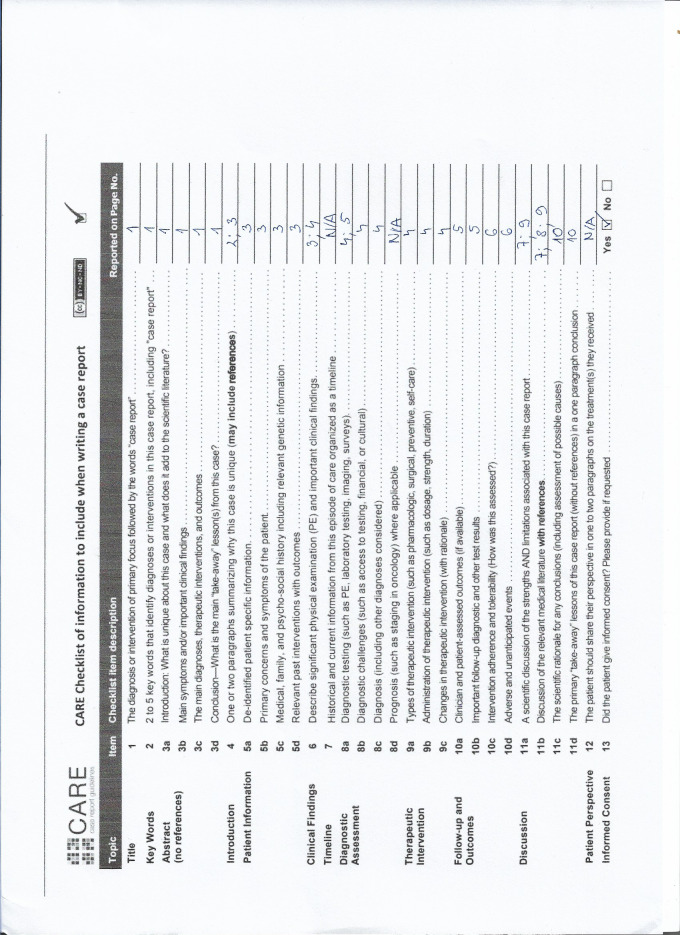# Calcifediol and paricalcitol as adjunctive therapies for HSV-1 keratitis and corneal perforation: A case report

**DOI:** 10.1097/MD.0000000000040654

**Published:** 2024-12-06

**Authors:** Vedran Nemet, Suzana Matić, Sarah J. Zielsdorf, Ivana Tolj, Marija Jelić Vuković, Luka Švitek, Miro Kalauz, Ivana Strunje, Lucija Matić, Marija Heffer

**Affiliations:** a Department of Ophthalmology and Optometry, Faculty of Medicine Osijek, J. J. Strossmayer University of Osijek, Osijek, Croatia; b Department of Ophthalmology, University Hospital Osijek, Osijek, Croatia; c Independent Researcher, West Chicago, IL; d Department of Internal medicine and History of medicine, Faculty of Medicine Osijek, J. J. Strossmayer University of Osijek, Osijek, Croatia; e Department of Nephrology, Clinic for Internal Diseases, University Hospital Osijek, Osijek, Croatia; f Department of Infectious Diseases and Dermatovenerology, Faculty of Medicine Osijek, J. J. Strossmayer University of Osijek, Osijek, Croatia; g Department of Infectious Diseases, University Hospital Osijek, Osijek, Croatia; h Department of Ophthalmology and Optometry, School of Medicine, University of Zagreb, Zagreb, Croatia; i Department of Ophthalmology, Zagreb University Hospital Center, Zagreb, Croatia; j Faculty of Dental Medicine and Health, J.J. Strossmayer University of Osijek, Osijek, Croatia; k Laboratory of Neurobiology, Department of Medical Biology and Genetics, Faculty of Medicine Osijek, J. J. Strossmayer University of Osijek, Osijek, Croatia.

**Keywords:** calcifediol, case report, corneal perforation, herpes simplex virus, paricalcitol, Vitamin D receptors

## Abstract

**Rationale:**

Herpes simplex virus 1 establishes a latent infection in trigeminal ganglia. Reactivation causes cold sores, as well as viral keratitis. The purpose of this study was to report potential benefits of using active vitamin D receptor ligands (VDR-agonists) as adjunctive therapies for the treatment of infectious corneal perforations, and prevention of HSV recurrence.

**Patient concerns:**

A 57-year-old female with a past history of episodic, poorly-healing, corneal erosions, recurring orolabial herpetic lesions, as well as PCR-confirmed recurrences of herpes simplex keratitis presented with a burning sensation and slight pain in the right eye. Examination indicated HSV keratitis. Topical antibiotic and oral antiviral treatments were prescribed. Despite these standard-of-care treatments, a perforated corneal ulcer ensued.

**Diagnoses:**

Corneal perforation associated with HSV-1 keratitis recurrence, later confirmed by PCR analysis of corneal scrapings.

**Interventions:**

Corneal perforation was treated with a human fibrin glue, fortified with multilayered amniotic membrane transplant, as well as a therapeutical contact lens. Following surgery, calcifediol and paricalcitol were started as oral adjunctive therapies in an attempt to boost tissue regeneration and innate-immunity within the slow-healing cornea. Anterior segment optical-coherence tomography was used to measure corneal thickness. Frequent follow-ups with various specialists allowed for comprehensive patient evaluation, and meticulous screening for any signs indicating potential HSV-1 recurrence.

**Outcomes:**

Following calcifediol-paricalcitol therapy corneal thickening, and re-epithelization ensued. During combined calcifediol-paricalcitol therapy, the patient has had no recurrence of herpes simplex keratitis, or orolabial herpes lesions.

**Lessons:**

Corneal stabilization avoided a high-risk, full-thickness corneal transplantation, facilitating future cataract surgery, and allowing for some degree of visual recovery in this eye.

## Graphical abstract

**Figure:**
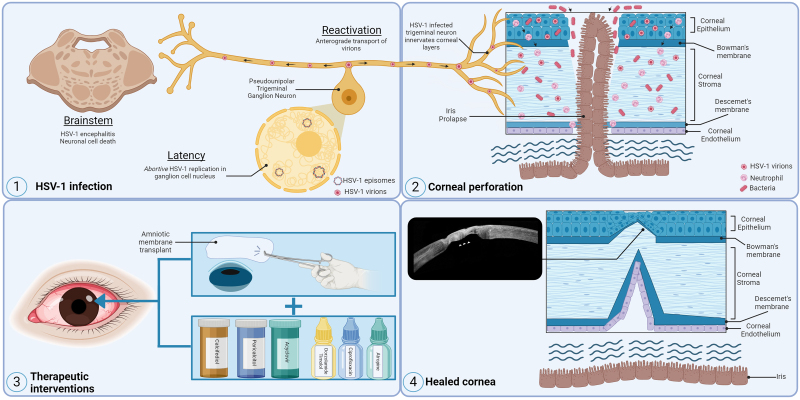

## 1. Introduction

Herpes Simplex Virus-1 (HSV-1) keratitis is the most common type of viral keratitis. Globally, the incidence of HSV-1 keratitis is 1.5 million yearly including 40,000 new cases that result in severe visual impairment.^[1]^ It is most commonly caused by recurrent HSV-1 reactivation within the sensory trigeminal ganglion.^[2]^ Some studies suggest that the cornea itself may also be a long-term reservoir, and not merely a transient site of HSV-1 infection emanating from trigeminal ganglia neurons.^[3]^ The process of abortive herpesvirus replication, in nonneuronal cells may help explain evidence that the cornea itself can be a tissue reservoir for HSV-1.^[4,5]^

Calcitriol (1,25-vitD3), the most active form of vitamin D, as well as calcifediol (25-vitD3) are both agonists of vitamin D receptors (VDRs). Paricalcitol (1,25-vitD2) is a synthetic, low-calcemic, VDR-agonist. Corneal epithelial and stromal cells contain VDRs as well as 1-α-hydroxylase, an enzyme responsible for converting calcifediol (25-hydroxycholecalciferol) into its active form calcitriol (1,25-dihydroxycholecalciferol).^[6–8]^

Calcitriol and its synthetic analogues, such as paricalcitol, have several potential beneficial effects which could slow or stop the progression of infectious keratitis, and corneal ulcer in advanced stages, as well as prevent perforation. They reduce the production of interleukin-8 and other chemokines which reduces corneal infiltration with polymorphonuclears, and associated collagen degradation.^[9,10]^ VDR-agonists also reduce the production of interleukin-1β, an important stimulus for the production of matrix-metalloproteinases (MMPs) in corneal fibroblasts. MMPs, especially MMP-2 and MMP-9, play a key role in collagen degradation during infectious keratitis.^[9–11]^ Reduction of interleukin-1β also contributes to the normal barrier function of corneal epithelial cells.^[12]^ 1,25-vitD3 upregulates the antimicrobial peptide cathelicidin LL-37 in cultured ocular tissue cells. The LL-37 peptide, produced by both corneal and conjunctival epithelial cells, among others, exhibits potent antibacterial and antiviral activity.^[13,14]^

In the paper, we report a case of HSV-1 keratitis that progressed to a corneal ulcer which eventually resulted in a corneal perforation. Oral 25-vitD3 supplementation, as well as oral 1,25-vitD2 were used as adjunctive therapies, to improve healing, re-epithelialization of corneal perforation, and reduce the risk of HSV keratitis recurrence.

## 2. Case presentation

**Figure 1.**
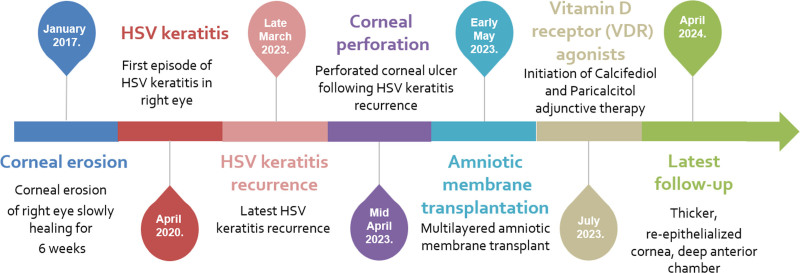
A timeline describing relevant ocular history, current symptoms, as well as therapeutical interventions used in this case report.

A 57-year-old female patient was referred to the emergency department due to keratitis in her right eye. Her ocular history was notable for a prior slowly-healing corneal erosion as well as previous polymerase chain reaction (PCR)-confirmed HSV-1 keratitis recurrences in the same eye (Fig. 1). The patient was regularly taking 40 mg of propranolol and 4 mg of perindopril daily for hypertension, but no other systemic, or ocular medications.

### 2.1. Previous herpes lesions and vaccination history

Prior to the onset of current keratitis, the patient had experienced orolabial herpes lesions several times a year over the past 10 years for which she was treated with episodic oral acyclovir (400 mg, 3 times daily for 5–7 days). She had never tested positive or had COVID-specific symptoms. The patient received her third and last dose of Pfizer mRNA vaccine at the end of 2021, 18 months prior to her infectious keratitis presentation.

### 2.2. Initial symptoms and early treatment

Initially, the patient reported a burning sensation in her right eye, as well as slight pain, redness, and photophobia lasting for 2 days. Her best-corrected visual acuity was 20/50 in the right eye, and 20/20 in the left eye. Intraocular pressure was 14 mm Hg in both eyes. Slit lamp evaluation of the right eye demonstrated conjunctival hyperemia. Corneal examination showed centrally located interstitial keratitis with epithelial defect, without neovascularization. There was also a slight stromal scaring in peripheral cornea from previously documented HSV keratitis recurrences. The anterior chamber of the right eye was deep with no inflammation. The lens was slightly opaque. Anterior segment examination of the left eye was unremarkable. Posterior segment examination was unremarkable in both eyes.

Due to the previous ocular history of herpes simplex keratitis, and frequent recurrences of orolabial herpes, the patient was started on oral acyclovir (400 mg, 5 times a day), topical 0.3% tobramycin drops (5 times a day), and 1% chloramphenicol ointment (2 times a day). Additionally, during alternate clinic visits, she received subconjunctival injections, each containing dexamethasone (1 mL, 4 mg/mL), gentamicin (1 mL, 60 mg/mL), and atropine (1 mL, 0.5 mg/mL).

Corneal scrapings were obtained under topical anesthesia and subjected to bacterial and fungal culture, but came back negative. Two days after initial presentation she had a negative PCR test for COVID-19. It is important to note that molecular diagnostic methods for ocular samples, such as PCR for HSV-1, were not available in our clinic at the time of presentation. Corneal scrapings were obtained, but results positive for HSV-1 were not received until almost 4 months after the initial presentation. The initial diagnosis of HSV keratitis recurrence was made based on clinical presentation as well as her past history of PCR-confirmed HSV-1 keratitis in the right eye (April 2020).

Despite treatment and regular follow-ups, corneal ulcer with stromal melting started developing after 4 days. Due to stromal melting and corneal ulcer formation, oral doses of the acyclovir were increased to 800 mg (5 times a day), topical 0.3% ofloxacin drops (hourly), 1% atropine (3 times a day), as well as 1% chloramphenicol ointment (2 times a day) were prescribed.

### 2.3. Corneal perforation: surgical treatments and outcomes

Ten days after her initial clinic visit (March 2023), the patient reported severe pain and decreased visual acuity of the right eye. Best-corrected visual acuity of the right eye was limited to hand motion. Slit lamp examination showed severe conjunctival hyperemia, a central perforated corneal ulcer with incarcerated iris, and a very shallow anterior chamber. The patient was hospitalized, and treated with oral acyclovir (800 mg, 5 times a day), oral ciprofloxacin (500 mg, twice a day), topical 0.3% ofloxacin (hourly), as well as a fixed combination of dorzolamide-timolol (2 times day), 1% atropine (3 times a day), and 1% chloramphenicol ointment (2 times a day).

During hospitalization corneal scrapings were obtained and cultured for bacteria, and fungus, but came back negative. Her PCR test for COVID-19 at this admission was also negative. A normal helper to cytotoxic T-cell ratio of 3.6 excluded a cellular immunodeficiency. Humoral immunodeficiency has also been excluded. Serology tests for syphilis and HIV were negative. Antinuclear factor, rheumatoid factor, extractable nuclear antigen panel, as well as C3 and C4 complement components were negative, and within normal ranges. A thorough neurological examination showed no signs of supratentorial or infratentorial lesions, brainstem or spinal cord involvement, nor signs of peripheral nerve pathology.

**Figure 2.**
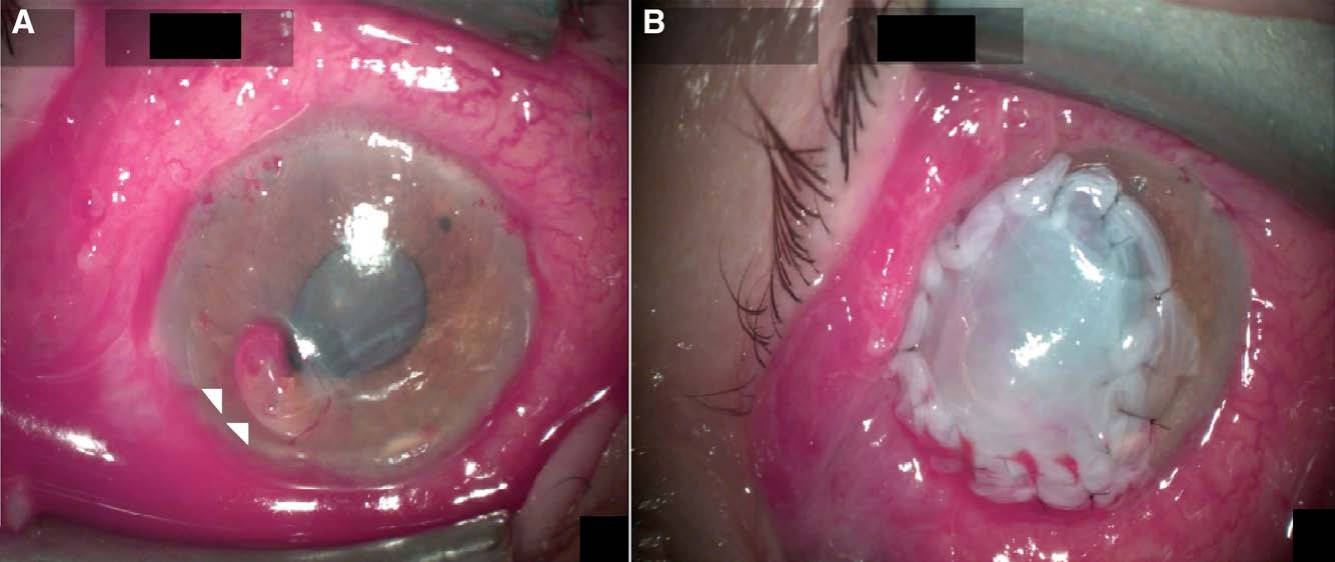
Surgical management of corneal perforation, following unsuccessful conservative treatment. (A) Intraoperative images of corneal perforation with iris pigment and viscoelastic leakage prior to amniotic membrane transplantation – marked by white arrowheads. (B) Multi-layered amniotic membrane transplanted over corneal perforation closed by fibrin glue.

Corneal perforation was treated with combination of human fibrin glue, and amniotic membrane transplant (AMT). A high-viscosity sodium hyaluronate viscoelastic was used to restore anterior chamber depth followed by a debridement of the ulcer. Human fibrin glue was applied directly on the corneal surface at the perforation site. The so-formed plug was then fortified with a multilayered AMT to avoid its extrusion. Viscoelastic was left in the anterior chamber to keep it formed, and prevent its shallowing following surgery. We believed washing it out immediately could extrude the glue plug. To avoid intraocular pressure spikes a fixed combination of dorzolamide-timolol was prescribed. A bandage contact lens was applied on top of AMT to prevent its displacement by blinking (Fig. 2). After 7 days of hospital treatment, there was gradual improvement in conjunctival inflammation and hyperemia so topical, and oral treatments were slowly tapered. Oral ciprofloxacin was stopped after 7 days, while acyclovir was reduced to 400 mg 5 times a day.

The patient was discharged from hospital after 4 weeks (May 2023) on topical therapy including 0.3% ofloxacin (3 times a day), a fixed combination of dorzolamide-timolol (2 times day), 1% atropine (3 times a day), 1% chloramphenicol ointment (2 times a day), and 100% autologous serum eye drops (every hour during daytime), as well as oral acyclovir (400 mg, 5 times a day). Oral acyclovir was discontinued 2 weeks after discharge.

Following complete absorption of the AMT, a thin, scared cornea, with epithelial defect, was observed at the site of prior perforation (June 2023). The anterior chamber was deep, and the iris was not incarcerated, indicating that the corneal perforation had closed. On subsequent follow-ups, slit lamp examination showed no epithelial regeneration. Anterior segment optical coherence tomography showed no increase in the thickness of the scared cornea for 8 weeks. Corneal thickness at the area of perforation was 90 µm, which only increased to 100 µm after 8 weeks, prior to calcifediol-paricalcitol (CP) therapy. This indicated delayed corneal healing and re-epithelization, as well as an increased risk for secondary bacterial infection and relapsed corneal perforation. A comprehensive literature analysis, indicated that VDR agonists could improve corneal healing based on cellular, and animal models so we decided to prescribe CP-therapy.

### 2.4. VDR-agonists used to promote corneal healing

Oral 25-vitD3 supplementation, combined with 1,25-vitD2was started to improve perforation-healing and corneal re-epithelialization reduce the probability of HSV-1 reactivation, as well as boost anti-microbial peptide production in ocular tissues. Both 25-vitD3 and 1,25-vitD2 are primarily used to treat secondary hyperparathyroidism in late-stage chronic kidney disease, and hemodialysis patients^[15,16]^ and both have been reported to provide substantial immune/anti-inflammatory benefits for renal patients.^[17]^

The patient discontinued oral acyclovir before starting 25-vitD3 and 1,25-vitD2 therapy. Laboratory values of intact PTH (parathyroid hormone), as well as ionized and total serum calcium were in normal ranges before initiating CP-therapy. The serum level of 25-vitD3 was 39 ng/mL which was within the normal range of serum 25-vitD3 levels (ref range: 30–100 ng/mL). The glomerular filtration rate of 97 mL/min/1.73m^2^ (July 2023) was age appropriate. She was started on oral 25-vitD3 (10 µg, twice daily), as well as oral 1,25-vitD2 (1 µg every other day) as an adjunctive therapy aimed at enhancing healing and re-epithelialization of the perforated cornea (July 2023). This 25-vitD3 dosing was in accordance with the recommended daily dose of a particular 25-vitD3 supplement (d.velop, Hologram Sciences).

**Figure 3.**
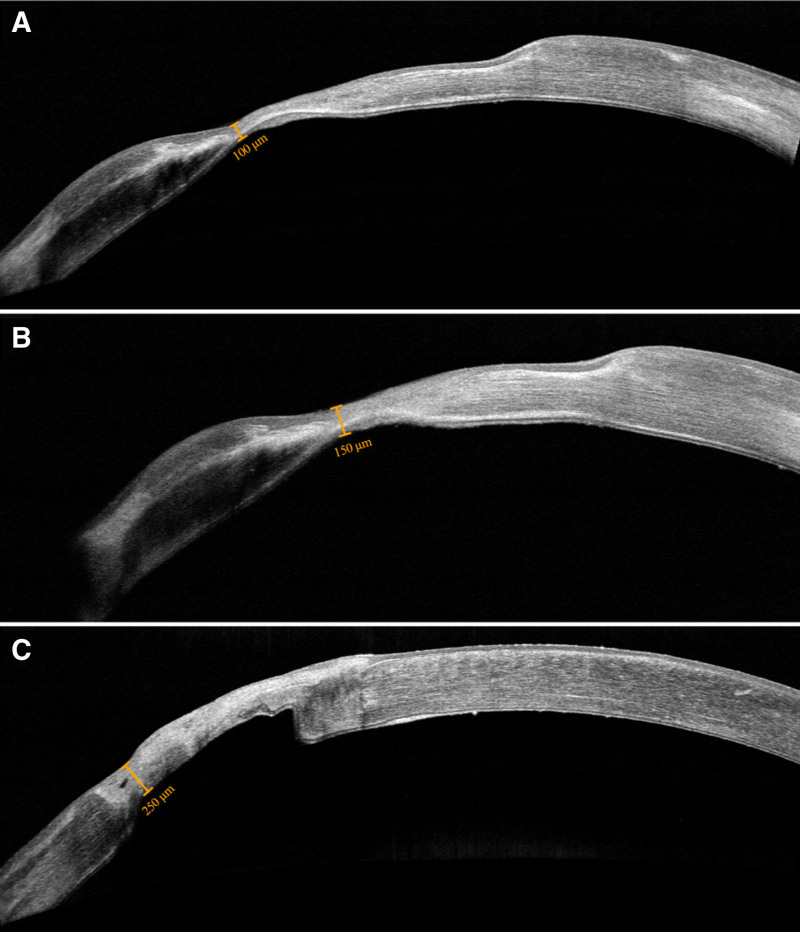
Anterior segment optical coherence tomography (OCT) images of patient’s cornea: (A) prior to calcifediol-paricalcitol (CP) therapy (July 2023), (B) after 1 month of calcifediol-paricalcitol (CP) therapy (August 2023), (C) after 3 months of calcifediol-paricalcitol (CP) therapy (October 2023).

The cornea was observed to thicken and began to re-epithelialize in the area of prior perforation allowing for a slow tapering of topical antibiotic therapy (Fig. 3). A therapeutic contact lens was applied and exchanged regularly. The 25-vitD3 (10 µg, twice daily) and 1,25-vitD2 (1 µg, every other day) were continued. Serum levels of 25-vitD3 increased to around 70 ng/mL within 4 weeks after the initiation of the above oral 25-vitD3 and 1,25-vitD2 supplementation (Table 1).

**Table 1:** Serum levels of electrolytes, vitamin D3 metabolites and parathyroid hormone

Date	April 2023	July 2023	August 2023	September 2023	November 2023	January 2024	April, 2024
Electrolytes (reference range)							
Calcium, ionized (1.18–1.32) mmol/L	Not measured	1.19	1.21	1.22	1.20	1.22	1.20
Calcium, total (2.14–2.55) mmol/L	Not measured	2.45	2.52	2.44	2.65	2.43	2.41
Phosphorus (0.79–1.32) mmol/L	Not measured	1.19	1.21	1.25	1.28	1.34	1.29
Renal function							
Blood urea nitrogen (2.8–8.3 mmol/L)	5.6	4.8	5.4	6.0	7.1	4.6	5.0
Creatinine (49–90) mmol/L	69	61	68	59	67	65	63
Vitamin D3 metabolites							
25D3 (30–100) ng/mL	Not measured	39	70	Not measured	82	59	65
Hormones							
PTH, intact (15–65) ng/L	Not measured	62	57	55	45	32	39
Calcifediol dose	None	None	20 µg daily	20 µg daily	20 µg daily	20 µg daily	20 µg daily
Paricalcitol dose	None	None	1 µg every other day	1 µg every other day	1 µg every other day	1 µg twice a week	1 µg twice a week

Boosted 25D3 serum levels following calcifediol-paricalcitol therapy, with serum calcium, phosphorus, and parathyroid hormone (PTH) levels within normal reference ranges. This illustrates the endocrine-mineral safety of calcifediol-paricalcitol therapy.

### 2.5. Corneal improvements with calcifediol-paricalcitol therapy

**Figure 4.**
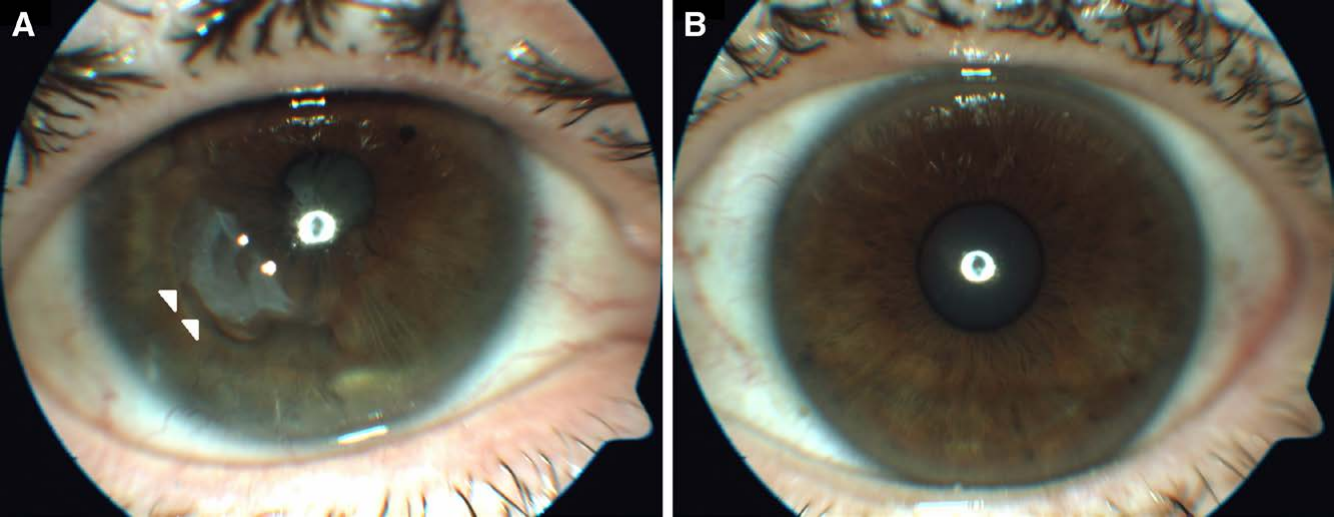
A calm, healing, anterior-segment following surgery, and subsequent CP-therapy. Anterior segment photographs of right (A) and left (B) eye on patient’s last visit, showing corneal thinning and opacification in the area of perforation – marked by white arrowheads.

On her latest follow-up, 1 year after the initial visit, the best-corrected visual acuity in the right eye was hand-motion, and 20/20 in the left eye. Slit lamp examination of the right eye showed quiet conjunctiva, early tear film breakup, central stromal opacification with thinning of the cornea, and superficial blood vessels, deep, formed, anterior chamber with dense cataract, as well as circumferential posterior synechiae (Fig. 4).

The patient currently uses artificial tears, oral 25-vitD3 (10 µg, twice daily), as well as oral 1,25-vitD2 (1 µg twice a week). Serum levels of 25-vitD3 reached 82 ng/mL (November 2023), and remained stable at 65 ng/mL (April 2024). Between July 2023 and late November 2023, she was taking 1,25-vitD2 (1 µg every other day). When her total serum Ca reached 2.65 mmol/L (2.55 mmol/L upper limit of normal serum Ca), 1,25-vitD2 dosing was lowered to 1 µg twice a week. Following this reduction, total serum Ca returned to normal ranges. The patient is monitored with regular laboratory tests and nephrologist follow-ups (Table 1).

Since the initiation of combined oral 25-vitD3 and oral 1,25-vitD2 (CP-therapy) in July 2023, the patient has had no recurrence of HSV-keratitis, or orolabial herpes lesions.

The CARE Checklist has been completed by the authors for this case report, attached as Supplementary Material, http://links.lww.com/MD/O80.

## 3. Discussion

Although the exact cause of corneal melting in this case was not confirmed at the time of perforation, the location and quick deterioration of corneal ulcer and perforation suggested some form of infectious keratitis. Late results of PCR analysis confirmed initial keratitis was caused by HSV-1 reactivation.

Possible past, and current, COVID infections in the patient were of concern, because COVID-19 infections can initially present with keratitis.^[18]^ In addition, latent HSV-1 can some-times reactivate in response to Covid vaccinations.^[19]^

Both 25-vitD3 and 1,25-vitD3 are immune-boosting, anti-inflammatory, exocrine-hormones, secreted in tear fluid by lacrimal glands.^[20]^ Chronic corneal inflammation disrupts a cranial sensory parasympathetic reflex circuit, causing deficient tear production, as well as a loss of 25-vitD3 and 1,25-vitD3 in tear fluid.^[21,22]^

Lu et al demonstrated that topical 1,25-vitD3 significantly accelerated corneal wound healing of normoglycemic, diabetic, and diabetic vitamin D deficient mice.^[23]^ Jabbehdari et al^[24]^ obtained similar results showing dose-dependent therapeutic effects of topical 1,25-vitD3 on corneal wound healing in vitro, on human corneal limbal-epithelial cells, as well as in vivo mouse corneas. Their research suggested that topical 1,25-vitD3 in a dose of 10–7 M (100 nM) significantly improved corneal wound healing time and reduced the expression levels of pro-inflammatory molecules such as ICAM1, TLR3, IL-6, IL-8, and TNFα when compared to other doses (10–6 M and 10–8 M), or vehicle.^[24]^

1,25-vitD2 has recently been found to suppress NETosis (NET = neutrophil extracellular trap) in activated neutrophils through upregulation of heme oxidase 1.^[25]^ NETosis contributes to corneal damage in infectious keratitis.^[26]^

Yin et al^[27]^ showed that both 25-vitD3 and 1,25-vitD3 increased corneal epithelial barrier function by increasing transepithelial resistance and reducing inulin permeability. Similarly, Elizondo et al demonstrated that 10-week-old VDR-knockout mice have reduced expression of occludin, a major protein component of the tight junction complex, compared to heterozygotes and wild-type mice.^[28]^ They also reported significantly decreased epithelium wound healing rate in 10-week-old VDR knockout mice compared to heterozygotes and wild-type mice.^[28]^

Lasagni Vitar et al^[29]^ demonstrated that oral vitamin D3 significantly reduced systemic collagen degradation in patients with keratoconus and vitamin D insufficiency (i.e., serum 25-vitD3 level). In their study, plasma levels of matrix metalloproteinase-9 (MMP-9) decreased, and levels of tissue metalloproteinase inhibitor-1 increased following oral vitamin D3 supplementation.^[29]^

Kumar et al^[30]^ showed that 25-vitD3 and 1,25-vitD3 supplementation reduced HSV-1 viral load and mRNA expression in HSV-1 infected HeLa cells. Similarly, Chao et al^[31]^ demonstrated that risk for reactivation of herpes zoster was significantly lower in hemodialysis patients who received vitamin D3 supplementation.

Human cathelicidin peptide LL-37, which is upregulated through VDR activation, has been found to inhibit replication of different viruses including HSV-1.^[32–34]^ LL-37 also inhibited pro-inflammatory cytokine release caused by stimulation of toll-like receptors 2,4, and 9 (TLRs 2,4, and 9).^[35]^ VDR agonists downregulated the expression of TLR2 and TLR4 in human monocytes.^[36]^ Bothou et al^[37]^ demonstrated potential benefits of anti-inflammatory and immuno-modulatory effects of 1,25-vitD3 and 1,25-vitD2 in the treatment of severe atopic dermatitis.

The primary risks associated with CP-therapy are hypercalcemia and hyperphosphatemia. Most cases of hypercalcemia with calcium levels < 3.0 mmol/L are asymptomatic.^[38]^ However severe hypercalcemia can affect gastrointestinal, renal, central nervous, cardiovascular, and musculoskeletal systems. When the levels of total serum calcium multiplied by levels of phosphorus exceed 4.85 mmol/L, calcium phosphate crystals start to accumulate within the soft tissues, possibly impairing renal function, and causing vascular calcification, as well as hypertension.^[39]^ Chowdry et al^[40]^ reported 19 cases of acute kidney injury induced by vitamin D toxicity with median serum levels of total calcium and 25-vitD3 at 3.25 mmol/L and 370 ng/mL, respectively. Long-term use of VDR-agonists has been utilized for osteoporosis treatment in post-menopausal women in China. It was shown to be well-tolerated, and without any significant side effects.^[41]^ Considering the stable renal function, mineral, and hormone levels, which lie within normal reference ranges (Table 1), as well as frequent nephrologist follow-ups, continuing her current immune-boosting CP-therapy appears to be well tolerated.

Currently, surgical techniques such as tissue adhesives, AMTs, and corneal patch grafts are used to treat imminent perforations as well as descemetoceles in patients with healed microbial keratitis. If these treatments prove ineffective, full-thickness corneal transplantation represents the final treatment option. This procedure carries a significant risk of both intraoperative, and postoperative complications. Prior microbial keratitis represents a high risk for graft rejection.^[42,43]^ The observed increase in corneal thickness, and improved re-epithelization in this patient, following CP-therapy, suggest that active VDR-agonists could serve as an adjunctive therapy to enhance the outcomes of initial surgical interventions. Consequently, reducing the requirement for full-thickness corneal transplantation in these patients with high risk for graft rejection.

Acyclovir (400 mg once or twice daily) is currently used for up to 1 year as a prophylaxis of herpes keratitis recurrence but with limited success. During the past year, the patient has had over 60 follow-up visits with 6 different specialists without any signs of skin, orolabial, or ocular herpes virus reoccurrence. This provides initial evidence that CP-therapy appears to be suppressing recurrence of herpes lesions. Vitamin D3 (cholecalciferol) supplementation, combined with fast-acting VDR-agonists, such as calcifediol and paricalcitol, could potentially replace acyclovir as a common prophylaxis of herpes virus reactivations.

In current ophthalmic clinical practice, doxycycline (100 mg, twice daily) is frequently used as an adjunctive therapy in treating infectious corneal ulcers and perforations for its inhibitory effect on matrix-metalloproteinases (MMPs), especially MMP-9.^[11]^ 1,25-vitD3 and other VDR-agonists exhibit similar effects, as discussed earlier, and can be used for prolonged periods without subjecting a patient to the toxicity and side effect of systemic antibiotic therapies. To potentially prevent corneal perforations, active VDR agonists should be started during initial corneal melting, and ulcer formation. We will consider CP-therapy in future patients with severe infectious keratitis during initial ulcer formation because we believe it could stop corneal stromal melting, and prevent perforation.

In the present case, there was a significant improvement in healing and re-epithelialization of the cornea, following the administration of CP-therapy as adjunctive therapies. It is possible this healing would have occurred without adjunctive CP-therapy, given enough time. However, thin, de-epithelialized cornea presents a significant risk factor for microbial keratitis recurrence as well as subsequent perforation.

Although current corneal thickness (290 µm) remains half of normal value, we consider this a positive outcome. Our primary goal in managing this perforation was to prevent endophthalmitis, and preserve the cornea. In the event of repeated corneal perforation, full-thickness corneal transplantation would be necessary. Given the high risk of graft rejection in patients with prior HSV-1 keratitis, the prospect of any visual recovery in this eye would be exceedingly challenging post-rejection. A thicker cornea facilitates potential cataract surgery in the future that could lead to some degree of visual recovery. We intend to continue CP-therapy until a planned cataract surgery, as well during her post-surgery recovery. CP-therapy may serve as a prophylaxis to reduce the probability of herpes virus reactivation in response to the planned cataract surgery.

### 3.1. Study limitations

This report contains several limitations. First limitation is the absence of molecular confirmation for HSV-1 keratitis at the time of initial presentation. Second limitation is a lack of direct correlation between calcifediol-paricalcitol therapy administration, and improved corneal healing. The effects of CP-therapy in this case are associated with, but not proven to be the only cause of the outcome. Additional studies are required where levels of calcitriol, calcifediol, and paricalcitol in tear fluid should be measured following their oral administration. Their tear fluid levels should be correlated with increase in corneal thickness, as well as reduction of inflammatory markers (such as MMP-9, LL-37, IL-6, IL-8, and TNF-α) in tears.

## 4. Conclusion

A stable closure of an HSV-1 keratitis-associated corneal perforation was promoted using calcifediol-paricalcitol (CP) therapy. Calcifediol and paricalcitol in this case led to improvement of initially slow corneal healing, remission of HSV-symptoms, as well as no evidence of HSV-1 recurrence. This post-surgery therapy involved boosting her serum 25-vitD3 to a sustained level around 60 to 80 ng/mL.

Following CP-therapy, corneal re-epithelization ensued, along with corneal thickening. As a consequence, full-thickness corneal transplantation was avoided in this patient with high risk for graft rejection. Thicker cornea facilitates cataract surgery in the future, allowing for some degree of visual recovery in this eye.

The absence of HSV-1 keratitis reoccurrence in this patient during CP-therapy, suggests that calcifediol and paricalcitol might be used as a general prophylaxis, to suppress recurring HSV-keratitis, as well as systemic HSV reactivation.

The current standard-of-care for corneal ulcers needs improvement. Corneal perforations could potentially be prevented through innate-immunity boosting as well as by the inhibition of corneal collagen degradation. In this paper, we have discussed the use of 2 adjunctive VDR-agonist therapeutics, calcifediol and paricalcitol which could potentially achieve this goal.

We hope the preliminary findings reported in this paper, will encourage further, comprehensive clinical research of the physiological actions of VDR-agonists on the cornea.

## Acknowledgments

We thank Dr Mark Cooper for suggesting paricalcitol as an immune-boosting therapeutic for our patient.

## Author contributions

**Conceptualization:** Vedran Nemet, Suzana Matić, Sarah J. Zielsdorf, Marija Heffer.

**Data curation:** Vedran Nemet, Suzana Matić, Ivana Tolj, Marija Jelić Vuković, Miro Kalauz, Ivana Strunje, Lucija Matić.

**Investigation:** Vedran Nemet, Suzana Matić, Ivana Tolj, Luka Švitek, Miro Kalauz, Lucija Matić.

**Resources:** Sarah J. Zielsdorf, Ivana Tolj, Luka Švitek, Marija Heffer.

**Supervision:** Sarah J. Zielsdorf, Miro Kalauz, Marija Heffer.

**Validation:** Vedran Nemet, Suzana Matić, Ivana Tolj, Luka Švitek, Marija Heffer.

**Visualization:** Marija Jelić Vuković, Ivana Strunje, Lucija Matić.

**Writing – original draft:** Vedran Nemet, Suzana Matić, Marija Jelić Vuković, Miro Kalauz, Ivana Strunje, Lucija Matić, Marija Heffer.

**Writing – review & editing:** Sarah J. Zielsdorf, Marija Jelić Vuković, Luka Švitek, Miro Kalauz, Ivana Strunje.

## Supplementary Material

**Figure:**